# A Role of the Lower Genital Tract Microbiome in Promoting Cervical Intraepithelial Neoplasia: A Premalignant Precursor of Cervical Cancer—A Literature Review

**DOI:** 10.3390/v18040403

**Published:** 2026-03-24

**Authors:** Weronika Knap-Wielgus, Agata Knap, Bronisława Pietrzak, Barbara Suchońska, Mirosław Wielgoś

**Affiliations:** 1 Doctoral School, Medical University of Warsaw, 02-091 Warsaw, Poland; 2 Department of Obstetrics and Perinatology, National Medical Institute of the Ministry of the Interior and Administration, 02-507 Warsaw, Poland; bronislawa.pietrzak@gmail.com (B.P.); miroslaw.wielgos@gmail.com (M.W.); 3 Maternal-Fetal Medicine Student Scientific Association, Medical University of Warsaw, 02-091 Warsaw, Poland; agataknap28@gmail.com; 4 1st Department of Obstetrics and Gynecology, Medical University of Warsaw, 02-015 Warsaw, Poland; barbara.suchonska@wum.edu.pl

**Keywords:** cervicovaginal microbiome, HPV, cervical intraepithelial neoplasia, cervical cancer, *Lactobacillus* species, community state types

## Abstract

The cervicovaginal microbiome (CVMB) is pivotal in maintaining the homeostasis of the lower female genital tract and has emerged as a significant modulator of cervical carcinogenesis. Although persistent infection with high-risk human papillomavirus (HR-HPV) is a prerequisite for the development of cervical intraepithelial neoplasia (CIN) and subsequent cervical carcinoma, it remains insufficient alone to drive oncogenesis. Accumulating evidence suggests that alterations in the CVMB composition profoundly impact HPV persistence, local immune responses, and disease progression. A vaginal microbiota dominated by *Lactobacillus* species, most notably *Lactobacillus crispatus*, correlates with low microbial diversity, robust immune regulation, and facilitated HPV clearance. Conversely, microbial dysbiosis—characterized by *Lactobacillus* depletion and a concomitant proliferation of anaerobic taxa, typical of Community State Type (CST) IV and *Lactobacillus iners*-dominated profiles—is strongly associated with chronic inflammation, oxidative stress, epithelial barrier compromise, and an elevated risk of CIN progression. This review synthesizes current evidence regarding the multifaceted interactions among the cervicovaginal microbiome, HPV pathogenesis, immune dysregulation, and oxidative stress in the etiology of CIN. Elucidating these intricate host–microbiome dynamics may precipitate the discovery of novel microbiome-derived biomarkers, ultimately informing innovative prophylactic and therapeutic interventions for cervical cancer.

## 1. Introduction

Over recent decades, the human microbiome has garnered intense scientific interest, with global research initially focusing predominantly on the gut microbiome. Its involvement in numerous immunological and oncological processes has been extensively documented. In contrast, the clinical significance of the vaginal microbiome—despite its profound impact on women’s health—emerged comparatively later. Unlike the intestinal microbiome, where high microbial diversity is indicative of a healthy state, the healthy lower female genital tract is typically dominated by one or a few *Lactobacillus* species [1,2]. Anatomically, the ectocervix is colonized by a distinct microbial community, whereas the endocervix and upper genital tract have traditionally been considered sterile in healthy women [2,3].

The Human Microbiome Project (HMP), initiated in 2008, marked the first large-scale endeavor to investigate the diversity of microorganisms across various anatomical niches of the human body. This landmark project analyzed microbiome composition, including that of the lower genital tract, in a cohort of approximately 300 healthy individuals [4]. In a broader oncological context, microbial agents are estimated to contribute to at least 16% of all malignancies worldwide, accounting for approximately two million cancer cases annually. Cervical cancer alone represents nearly 50% of this infection-associated cancer burden among women [5,6]. It is unequivocally established that the principal etiologic agent driving both cervical intraepithelial neoplasia (CIN) and cervical carcinoma (CC) is persistent infection with the human papillomavirus (HPV), most notably high-risk oncogenic genotypes such as HPV 16 and 18 [7].

Microorganisms play a central role in shaping the lower genital tract environment by modulating its biochemical and inflammatory properties. Consequently, alterations in this environment may influence not only genital symptoms but also fertility, pregnancy outcomes, susceptibility to sexually transmitted infections (STIs), and the risk of developing precancerous and, subsequently, malignant lesions of the vagina and cervix [8]. This latter aspect constitutes the primary focus of the present review. Recent studies increasingly suggest that the cervicovaginal microbiota (CVMB) may act as an important cofactor in the etiology of cervical cancer and its precursor, cervical intraepithelial neoplasia [9,10,11]. These findings indicate that CVMB composition may serve as a valuable biomarker for disease risk stratification and, potentially, as a tool to inform future therapeutic and preventive strategies.

## 2. Materials and Methods

This review aims to synthesize current findings on the role of microbiome of the lower genital tract in promoting cervical intraepithelial neoplasia. To support this synthesis, in January 2026, an extensive literature search was conducted in PubMed/MEDLINE using the following Boolean search strategy: (“cervicovaginal microbiome”[tiab] OR “vaginal microbiome”[tiab] OR “cervicovaginal microbiota”[tiab] OR “vaginal microbiota”[tiab]) AND (“HPV”[tiab] OR “human papillomavirus”[tiab] OR “Papillomaviridae”[Mesh]) AND (“cervical intraepithelial neoplasia”[tiab] OR “Cervical Intraepithelial Neoplasia”[Mesh] OR “cervical cancer”[tiab] OR “CIN”[tiab]).

Articles in languages other than English were excluded. We primarily included peer-reviewed studies published between 2000 and 2025, covering systematic reviews, meta-analyses and original research—including preclinical animal model studies, as well as prospective, randomized, controlled, and uncontrolled clinical trials.

## 3. The CVMB and Its Function

Based on the seminal analysis of the vaginal microbiota in 396 women of diverse ethnic backgrounds, five distinct community state types (CSTs) of the cervicovaginal microbiome (CVMB) have been delineated [12]. The species composition were characterized by pyrosequencing of barcoded 16S rRNA genes. These CSTs are primarily distinguished by the species composition and relative abundance of *Lactobacillus*, a keystone genus essential for vaginal eubiosis. Community State Type I (CST I) is widely regarded as the most optimal state, predominantly characterized by *Lactobacillus crispatus*. Similarly, CST II, CST III, and CST V represent healthy states, dominated by *Lactobacillus gasseri*, *Lactobacillus iners*, and *Lactobacillus jensenii*, respectively. Conversely, CST IV is characterized by a marked depletion of lactobacilli and a concomitant proliferation of anaerobic and microaerophilic taxa, including *Prevotella*, *Dialister*, *Atopobium*, *Gardnerella*, *Megasphaera*, *Peptoniphilus*, *Sneathia*, *Eggerthella*, *Aerococcus*, *Finegoldia*, and *Mobiluncus*. This specific community state notably lacks a single dominant bacterial species. Community State Types (CSTs) of the cervicovaginal microbiome (CVMB) are presented in Table 1.

The dominance of lactobacilli within the vaginal ecosystem is widely believed to be driven primarily by glycogen availability, which accumulates in the cervicovaginal epithelium in response to estrogen stimulation [8]. *Lactobacillus* species, the hallmark of a healthy microbiome, utilize this glycogen as a primary energy source. Both human and bacterial enzymes, such as alpha-amylase and other glycogen-degrading enzymes (GDEs) are involved in breaking down complex glycogen into simpler sugars (maltose, maltotriose) that *Lactobacillus* can consume. More importantly, *Lactobacillus* species metabolize the glycogen into lactic acid, which is critical for creating an acidic environment. The resulting low pH (3.5–4.5) inhibits the growth of strict and facultative anaerobes (e.g., *Gardnerella vaginalis*) that thrive in higher, more alkaline pH, thus preventing bacterial vaginosis.

In a healthy female genital tract, lactobacilli inhibit colonization by pathogenic microorganisms through the production of lactic acid and bacteriocins, thereby preserving mucosal barrier integrity and protecting against viral and opportunistic bacterial infections [13,14,15]. Lactic acid exists in two isomeric forms—D-lactic and L-lactic acid—of which D-lactic acid exhibits significantly stronger protective properties against infection. Most *Lactobacillus* species produce D-lactic acid. Although *Lactobacillus* species are predominant in the cervicovaginal environment of most women and contribute to maintenance of a low vaginal pH, individual species may play distinct roles in cervical carcinogenesis [16]. Notably, *L. iners* differs from other lactobacilli in possessing a relatively small genome and lacking the ability to produce D-lactic acid. Instead, it produces L-lactic acid, which has been associated with an increased risk of viral infections in several studies. Moreover, *L. iners* can secrete inerolysin, a cholesterol-dependent cytolysin similar to vaginolysin produced by *Gardnerella*, which disrupts epithelial integrity and increases susceptibility to viral infections [17].

The role of hydrogen peroxide produced by *Lactobacillus* species remains controversial. On one hand, H_2_O_2_ has been attributed antimicrobial and potentially antitumor properties, particularly in the presence of peroxidases and halide ions, which facilitate the formation of hypochlorous acid (HOCl). However, more recent experimental studies strongly question this thesis, pointing to the lack of H_2_O_2_ efficacy under actual in vivo conditions [18]. A study by O’Hanlon et al. demonstrated that physiological concentrations of H_2_O_2_ (<100 μM) do not inactivate any of the analyzed bacteria associated with bacterial vaginosis (BV) [19]. In fact, a concerning paradox was observed: at very high concentrations, hydrogen peroxide proved to be more toxic to the beneficial lactobacilli (*Lactobacillus*) themselves than to the bacteria causing the infection. Another study by the same author showed that cervicovaginal fluid (CVF) and semen possess strong H_2_O_2_-neutralizing and blocking properties [20]. The addition of merely 1% of vaginal fluid supernatant was enough to completely abolish the in vitro antimicrobial activity of hydrogen peroxide-producing lactobacilli. Furthermore, the vaginal environment is typically hypoxic by nature, whereas *Lactobacillus* bacteria strictly require oxygen to produce hydrogen peroxide, meaning that its in vivo production under the anaerobic conditions of the vagina is virtually impossible [21]. Increasing evidence suggests that lactic acid plays a more critical protective role, as its production is enhanced under anaerobic conditions and it effectively inhibits the growth of pathogens and sexually transmitted infections. Lactic acid has also been shown to inactivate bacteria associated with bacterial vaginosis as well as *Chlamydia trachomatis*, an effect not observed for hydrogen peroxide [19,22]. Additionally, a balanced vaginal microbiota is associated with increased levels of defensins—antimicrobial peptides produced in the vagina that prevent pathogen adhesion to epithelial cells. Consistently, women with bacterial vaginosis exhibit reduced concentrations of these peptides.

The cervicovaginal environment is dynamic, with microbiome composition undergoing physiological fluctuations throughout a woman’s life. Sex hormone levels—particularly estrogen—modulate vaginal glycogen content and pH, allowing microbial communities to adapt to changing metabolic and immunological demands. Consequently, CVMB composition varies across the menstrual cycle, pregnancy, lactation, and menopause [23]. During the follicular and ovulatory phases, elevated estrogen levels promote glycogen accumulation, favoring *Lactobacillus* dominance and lower vaginal pH. In contrast, the luteal phase and menstruation are associated with reduced estrogen levels and increased microbial diversity, accompanied by a relative decrease in lactobacilli abundance [24]. The use of oral contraceptives or intrauterine systems has not been consistently associated with significant changes in microbiome composition or diversity [24]. During pregnancy, the vaginal microbiota is typically dominated by *Lactobacillus species*—particularly *L. crispatus* and *L. iners*—and is characterized by low microbial diversity. Following childbirth, a pronounced shift occurs, with decreased lactobacilli and increased microbial diversity, including anaerobic taxa commonly associated with CST III and CST IV profiles, where *L. iners* often becomes predominant. These communities are generally less stable and more diverse than those dominated by other lactobacilli. Hormonal changes during lactation and menopause, especially declining estrogen levels, further reduce lactobacilli dominance, increase vaginal pH, and promote microbial diversity, potentially heightening susceptibility to infection and adversely affecting urogenital health [23,25].

In addition to hormonal influences, CVMB composition is shaped by multiple factors, including ethnicity, sexual activity, hygiene practices, metabolic disorders such as diabetes mellitus, stress, and dietary habits. Numerous studies have demonstrated ethnic differences in vaginal microbiome composition, with African American and Hispanic women more frequently exhibiting *non-Lactobacillus-*dominan*t* communities. Importantly, these microbiome profiles are more often associated with bacterial vaginosis and an increased risk of sexually transmitted infections, suggesting that racial and ethnic differences in CVMB composition may partially explain disparities in disease prevalence [25,26,27,28]. The underlying reasons for ethnic differences are multifactorial, involving a combination of genetics, biology, and environmental, social, and behavioral factors. Genetic factors likely influence the vaginal environment, determining which bacteria can thrive. Differences in innate and adaptive immunity, which shape the microbiome, may be rooted in genetic diversity. Moreover, differences in vaginal cleaning practices (e.g., douching), hormonal levels, sexual activity, contraceptive use, smoking status, and dietary habits are linked to ethnicity and influence the microbiome. Factors such as lower socioeconomic status and higher stress levels—which disproportionately affect some racialized communities—have been associated with higher incidences of bacterial vaginosis and less *Lactobacillus-*dominant microbiomes. Recent research suggests that host–microbe interactions and evolutionary processes—such as adaptations to specific environments—have led to distinct, stable vaginal microbial communities across different populations. It is important to note that these differences do not mean that one type is inherently “unhealthy.” Instead, they suggest that what constitutes a “healthy” vaginal microbiome can vary by ethnicity [29,30,31].

## 4. The Correlation Between CVMB and CIN

**Figure 1:**
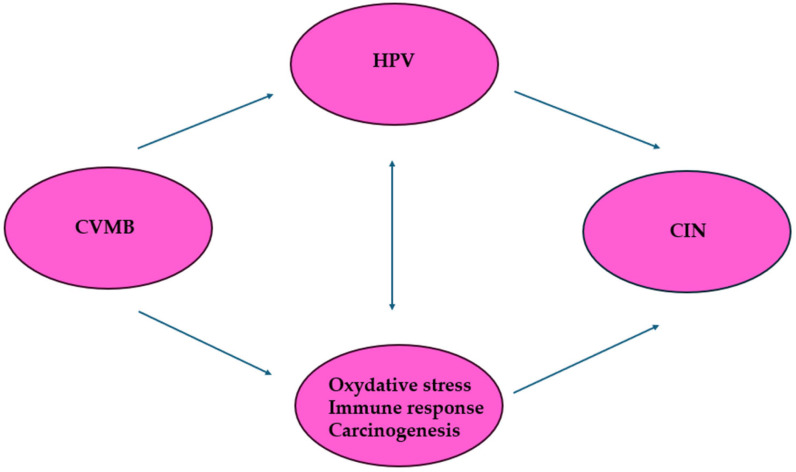
Two possible paths to explain the association between CVMB and CIN.

A growing body of literature indicates that the cervicovaginal microbiome (CVMB) is fundamentally implicated in the development of precancerous and cancerous cervical lesions, acting both through direct modulation of carcinogenic processes and by indirectly facilitating HPV infection [16]. These dual pathways of CIN pathogenesis are illustrated in Figure 1. While persistent high-risk HPV (HR-HPV) infection is an indispensable prerequisite for the development of cervical intraepithelial neoplasia (CIN), it remains insufficient as a solitary driver of oncogenesis. Substantial evidence underscores distinct taxonomic variations in CVMB composition across women with normal cytology, HR-HPV infection, and CIN.

For instance, Mitra et al. elucidated that advancing CIN severity correlates inversely with the relative abundance of *Lactobacillus* species. In their cohort study of 169 women, the distribution of Community State Types (CSTs) was evaluated among individuals with normal cytology (control group), low-grade squamous intraepithelial lesions (LSIL), high-grade squamous intraepithelial lesions (HSIL), and invasive cervical cancer (ICC). CST I, characterized by the dominance of *Lactobacillus crispatus*, was most prevalent in the control group. In stark contrast, CST IV—defined by *Lactobacillus* depletion and profound microbial diversity—was observed twice as frequently in the LSIL cohort, three times more often in the HSIL group, and up to four times more frequently in patients with ICC compared to normocytological controls [32]. Corroborating these findings, a Polish cohort study by Kwaśniewski et al. (*n* = 250) demonstrated analogous trends. Among 70 HPV-negative participants with normal cytology, the vaginal microbiome exhibited high abundances of *L. crispatus*, *L. iners*, and *L. taiwanensis*, concurrent with an absence of *Gardnerella vaginalis* and *Lactobacillus acidophilus*. In HPV-positive women with LSIL (*n* = 95), *L. crispatus* was significantly diminished relative to controls, whereas *L. acidophilus* and *L. iners* predominated. Conversely, within the HPV-positive HSIL cohort (*n* = 95), a marked enrichment of *G. vaginalis* and *L. acidophilus* was observed, accompanied by significantly reduced frequencies of *L. iners*, *L. crispatus*, and *L. taiwanensis* compared to the control group [33]. Furthermore, a Chinese study profiling the cervicovaginal microbiota of 60 women with CIN and 60 healthy controls revealed that the microbiomes of CIN patients were significantly enriched in the genera *Sphingomonas* and *Stenotrophomonas*. Multivariate analysis further identified *Comamonas*, *Rhizobium*, and *Pseudomonas* as independent genera contributing to the progression of cervical dysplasia [34].

Further investigations have explored the prognostic value of the CVMB in the spontaneous regression of precancerous cervical lesions. A longitudinal study by Mitra et al. monitored 87 women with untreated CIN2 over a 24-month follow-up period. The authors demonstrated that patients harboring a *Lactobacillus*-dominant microbiota exhibited a significantly higher probability of lesion regression at 12 months. Conversely, the depletion of *Lactobacillus* species and the colonization by anaerobic taxa—such as *Megasphaera*, *Prevotella timonensis*, and *G. vaginalis*—were robustly associated with CIN2 persistence and delayed regression. By the conclusion of the two-year follow-up, CIN2 regression was documented in 81% of the retained cohort [35]. These findings strongly imply that a eubiotic vaginal microbiome not only buffers against abnormal cervical epithelial responses and pathogen infiltration, including HPV, but may also actively orchestrate lesion regression and viral clearance.

While the majority of research has historically focused on isolated correlations—either between the microbiome and HPV infection, or between the microbiome and CIN development—Zhang et al. (2018) comprehensively analyzed both the direct oncogenic effects of specific bacterial taxa and their indirect effects mediated via HPV infection [16]. This cross-sectional study (*n* = 166) stratified patients with normal cytology, CIN1, CIN2, and CIN3 by HPV infection status. HPV prevalence was recorded at 10.9% in women with normal cytology (*n* = 64), 12.9% in CIN1 (*n* = 62), 31.6% in CIN2 (*n* = 19), and 71.4% in CIN3 (*n* = 21). The analysis revealed that bacteria such as *Pseudomonas stutzeri*, *Bacteroides fragilis, Lactobacillus delbrueckii, Atopobium vaginae*, and *Streptococcus agalactiae* may drive CIN pathogenesis indirectly by facilitating HPV infection. Notably, *P. stutzeri* was entirely absent in HPV-negative CIN groups, suggesting it lacks a direct mechanistic role in CIN formation. In contrast, *A. vaginae* was detected in HPV-negative CIN cases, indicative of a potential dual (direct and indirect) role in cervical carcinogenesis. The remaining taxa—*B. fragilis*, *L. delbrueckii*, and *S. agalactiae*—were implicated in CIN progression exclusively through indirect, HPV-mediated mechanisms [16].

## 5. The Interplay Between CVMB and HR-HPV Infection in the Process of CIN Forming (Indirect Pathway)

The lifetime risk of acquiring any type of HPV infection is estimated to be as high as 80%. While the majority of immunocompetent women spontaneously clear the virus, approximately 10–15% of HR-HPV infections persist. These persistent infections can instigate the development of precancerous cervical intraepithelial neoplasia (CIN) and, if left untreated, progress to invasive cervical carcinoma (ICC), carrying an estimated progression risk of approximately 0.6% [2,36]. The exact mechanisms dictating why certain HR-HPV infections resolve while others precipitate dysplastic progression and malignancy remain incompletely elucidated. To date, several cofactors—including tobacco smoking, sexual and reproductive behaviors, and immunosuppressive conditions such as human immunodeficiency virus (HIV) infection—have been established as promoters of HPV persistence [37]. Recently, multiple small-scale investigations have posited a strong association between alterations in the cervicovaginal microbiota composition and HPV infection dynamics, though earlier meta-analyses frequently failed to resolve these associations at the species level [10,11,38]. The current evidence suggests that women harboring a highly diverse vaginal microbiota may face an elevated risk of HPV acquisition or accelerated dysplastic progression, thereby necessitating more stringent clinical surveillance or aggressive therapeutic interventions [39]. Furthermore, an ecological shift favoring pathogenic microorganisms over lactic acid-producing bacteria is thought to critically impair viral clearance and promote HPV persistence [32,40,41,42,43].

A comprehensive meta-analysis conducted by Norenhag et al. demonstrated that community state types defined by *Lactobacillus* depletion (CST IV) or *Lactobacillus iners* dominance (CST III) were correlated with a significantly higher prevalence of HPV compared to *Lactobacillus crispatus*-dominated communities (CST I) [39]. This association held true for both HR-HPV infections and overall HPV prevalence, with analogous trends documented for cervical dysplasia and invasive cancer. Interestingly, one longitudinal study indicated that *Lactobacillus gasseri*-dominated communities (CST II) were associated with the most rapid rates of HPV clearance [16]. Earlier cross-sectional analyses involving a Korean twin cohort (*n* = 68) and a Chinese cohort (*n* = 70) revealed that HPV-positive women without concurrent cervical dysplasia exhibited markedly greater vaginal microbial diversity. These individuals also harbored significantly higher abundances of *L. gasseri* alongside bacterial vaginosis (BV)-associated taxa, including *Gardnerella*, *Sneathia*, *Megasphaera*, and *Dialister*, when compared to their HPV-negative counterparts [35,43].

Corroborating this, a subsequent network meta-analysis incorporating both cross-sectional and longitudinal data revealed that women with a vaginal microbiome either lacking *Lactobacillus* dominance or dominated by *L. iners* faced a three- to fivefold higher probability of any HPV infection. Moreover, these profiles conferred a two- to threefold increased risk of HR-HPV infection and cervical neoplasia relative to women with *L. crispatus*-dominated microbiomes. Notably, women exhibiting *L. gasseri*-dominated microbiota demonstrated the highest risk of HR-HPV infection relative to the *L. crispatus* baseline. These epidemiological trends were validated by an independent meta-analysis, which confirmed that *L. crispatus* dominance is robustly protective against HR-HPV detection and cervical dysplasia, an effect notably absent in *L. iners*-dominated profiles [9].

In a targeted cross-sectional cohort of 117 women with documented HPV status, CST IV was significantly overrepresented among HR-HPV-positive individuals compared to their HR-HPV-negative peers. Conversely, HPV-negative women predominantly exhibited CST I profiles, whereas the distribution of other CSTs did not reach statistical significance between the cohorts [32]. Aligning with these observations, a longitudinal analysis of vaginal swabs collected over a 16-week period from 32 sexually active women demonstrated that a CST IV microbiome—characterized by profound *Lactobacillus* depletion and *Atopobium* enrichment—correlated with the most protracted rates of HPV clearance. In contrast, CST II communities dominated by *L. gasseri* were predictive of the most rapid viral regression [44]. Although limited by sample size and statistical power, this study underscored the critical utility of longitudinal microbiome profiling in mapping the temporal dynamics between CVMB fluctuations and HPV pathogenesis. Furthermore, a study by Di Paola et al. identified specific anaerobic taxa, including *Gardnerella*, *Prevotella*, *Atopobium*, and *Sneathia*, as distinct independent biomarkers for HR-HPV persistence [45].

## 6. The Interplay Between CVMB, Immune Response and Oxidative Stress in the Process of CIN Forming (Direct Pathway)

Vaginal dysbiosis, chronic inflammation, and oxidative stress are closely linked in a detrimental cycle that promotes the persistence of HR-HPV and the progression to cervical cancer. A healthy vaginal microbiome is dominated by *Lactobacillus* species, which maintain a low pH and prevent infection. When this balance is lost (dysbiosis), harmful bacteria thrive, causing chronic inflammation and oxidative stress, which aid in HPV survival and DNA damage. In humans, the cervicovaginal microbiome (CVMB) closely interacts with the local tissue environment to preserve physiological homeostasis. Disruption of this equilibrium, referred to as dysbiosis, may initiate a range of pathological mechanisms, including the loss of epithelial barrier integrity, uncontrolled cell proliferation, genomic instability, enhanced angiogenesis, persistent inflammation, and disturbances in metabolic regulation. Ultimately, these processes may lead to carcinogenesis and, in the context of the CVMB, to the development of cervical intraepithelial neoplasia (CIN) and invasive cervical cancer.

Oxidative stress is an important factor linking the disruption of the lower female genital tract microenvironment with carcinogenesis. The excessive production of reactive oxygen species (ROS)—such as the superoxide anion, hydroxyl radical, and hydrogen peroxide—leads to the damage of proteins, lipids, and DNA. In the context of HPV infection, oxidative stress promotes the integration of the viral genome into the host DNA, the dysregulation of the *E2* gene, and the uncontrolled activation of the *E6* and *E7* oncogenes, resulting in increased cellular proliferation and the inhibition of apoptosis [46,47,48,49,50,51]. Chen et al. confirmed the interplay between oxidative stress and vaginal microbiota composition [52]. Their study demonstrated that hydrogen peroxide (H_2_O_2_) levels in women with bacterial vaginosis were nearly tenfold higher than in healthy individuals, indicating the presence of oxidative stress in this group [52]. This corroborates the previously mentioned adverse role of hydrogen peroxide in the cervicovaginal environment.

Disturbances in the vaginal microbiome, particularly bacterial vaginosis (BV), are closely associated with the induction of local inflammation in the lower genital tract. A key mechanism underlying this process is the reduction in lactic acid concentration, resulting in the loss of its anti-inflammatory properties. Under physiological conditions, lactic acid inhibits Toll-like receptor (TLR) activation and promotes the production of the interleukin-1 receptor antagonist (IL-1Ra), thereby limiting inflammatory responses. In contrast, metabolites characteristic of BV enhance the production of pro-inflammatory cytokines, such as TNF-α, while simultaneously reducing the levels of chemokines and proteins involved in antiviral immune responses [28,53].

A vaginal environment lacking *Lactobacillus* dominance is characterized by elevated levels of pro-inflammatory, chemotactic, and regulatory cytokines, which correlate with dysbiosis, chronic inflammation, and the development of dysplastic lesions and cervical cancer. Population-based studies have shown that high bacterial diversity, including taxa such as *Sneathia, Prevotella, Fusobacterium*, and *Mobiluncus*, is associated with the increased expression of pro-inflammatory cytokines (including IL-1α, IL-1β, IL-8, IL-36γ, and TNF-α), chemotactic cytokines (IP-10, MIP-1β, and RANTES), hematopoietic factors (FLT3 ligand), and mediators of adaptive immunity (IL-2, IL-4, and soluble CD40 ligand), both in vitro and in vivo [26,54]. It has been suggested that lipopolysaccharides (LPS) present in the outer membrane of Gram-negative bacteria may be recognized by antigen-presenting cells (APCs) within the cervical mucosa, leading to the activation of Toll-like receptor 4 (TLR4), the initiation of the NF-κB signaling pathway, and the increased production of pro-inflammatory cytokines and T-cell-recruiting chemokines. In women with a high abundance of *Prevotella* species, enhanced immune responses to lipopolysaccharides, interferon-γ (IFN-γ), and interleukin-1β (IL-1β) have been observed, likely reflecting a host defense response to Gram-negative microorganisms. Additionally, in microbiota communities classified as CST IV, APCs exhibit increased expression of CD80, ICAM-1, and MHC class II molecules, thereby promoting T-cell activation and enhancing their effector functions [45]. This mechanism appears to explain why, as a result of T-cell activation in CST IV and HPV infection, the condition does not always progress to a persistent cervical infection and high-grade CIN. The activation processes of this defense pathway in a dysbiotic environment simply take longer than in a healthy vaginal environment rich in Lactobacillus species, which can eliminate the HPV infection more rapidly and effectively without triggering these extensive immune pathways. For that reason, the lack of T-cell immunity, observed, for example, in individuals with AIDS, significantly increases the risk of cervical cancer and its precancerous lesions [55].

Alterations in the cervicovaginal microbiome may also interact with HPV infection, playing an important role even at the early stages of carcinogenesis. In a study by Audirac-Chalifour et al. [56], the composition of the cervical microbiome and cytokine profiles were analyzed across successive stages of cervical cancer development, leading to the proposal of a model of microbiota changes induced by infection with oncogenic HPV types. HPV infection was associated with a shift from *Lactobacillus crispatus* dominance to *Lactobacillus iners*, followed—along with the progression to squamous intraepithelial lesions (SIL) and cervical cancer—by increased microbial diversity, particularly involving bacteria of the genera *Sneathia* and *Fusobacterium*. The presence of *Fusobacterium necrophorum* in cervical cancer further amplified this diversity. The authors suggest that HPV infection promotes the formation of an immunosuppressive microenvironment characterized by increased IL-10 expression, macrophage polarization toward the M2 phenotype, and enhanced microbiota-derived TGF-β1 signaling, thereby creating a positive feedback loop between microbiota composition and cytokine profiles. In addition, a *Fusobacterium*-dominated microbiota is associated with a predominance of Th2-type immune responses, elevated levels of IL-4 and TGF-β1, and the disruption of the E-cadherin/β-catenin signaling pathway in HPV-transformed cervical epithelial cells [56]. 

## 7. Probiotics as a Therapeutic Option for HPV, Cytological Changes, and Cervical Cancer

Probiotics from the *Lactobacillus* genus are attracting broad attention as a promising, non-pharmacologic therapeutic alternative for cervical cancer, HR-HPV infections, and precancerous changes. *Lactobacillus* species can not only acidify the vaginal environment, stabilize the vaginal flora, and support vaginal epithelial cell function, but they may also destroy cervical cancer cells. *Lactobacilli* adhere to and colonize the vaginal epithelium, preventing the adherence of aggressive pathogenic bacteria that drive malignancy [49]. Furthermore, *Lactobacillus* may inhibit cancer cell proliferation by secreting peptidoglycans and exopolysaccharides. Probiotics primarily enhance the body’s immune processes, promote cytokine production, and inhibit monocyte proliferation. Recent studies indicate that probiotics such as *Lactobacillus casei* and *Lactobacillus rhamnosus* play an anticancer role by activating the maturation of natural killer (NK) cells and dendritic cells [57,58]. Additionally, *Lactobacillus* may influence cellular and humoral immunity, promote the proliferation and differentiation of thymus-derived cells, and further support immunological recognition and the proliferation of bone-marrow-derived cells [59]. The metabolites of probiotics also have cytotoxic effects on cervical cancer cells.

The rationale for administering oral lactobacilli is based on the interconnection between the gut and vaginal microbiomes, suggesting that much of vaginal dysbiosis may originate in the intestine [14,60]. T. Wang et al. compared the gut microbiota of eight women with cervical cancer to that of five healthy women [60]. They found that those with cervical cancer had greater gut microbiota diversity, although this difference did not reach statistical significance. Conversely, there has been a growing interest in vaginal microbial transplantation (VMT) in recent years [61,62]. One study investigated the effect of VMT on vaginal dysbiosis using an established model [63]. The results demonstrated that VMT significantly reduced bacteria-induced inflammation and the enrichment of pro-inflammatory cytokines, successfully restoring normal vaginal microbiota.

A prospective, controlled pilot study by Liu et al. assessed the effects of probiotics on cervical cytology changes and HPV infection [64]. The trial recruited 100 women with high-risk HPV infections, randomly dividing them into a placebo group and a treatment group that received intravaginal transplantations of *L. crispatus* chen-01. After six months, cervical exfoliated cells were collected to detect DNA load, type the HPV, and perform cytological analysis. The results showed that vaginal transplantation with *L. crispatus* chen-01 significantly reduced the HR-HPV viral load and improved the clearance rate. The total effective HPV clearance rate in the probiotic group was 12.13% higher than in the placebo group. Furthermore, the cytological improvement rate was 82.14% for the probiotic group compared to 34.62% for the placebo group [64].

In an open, non-controlled study by Di Pierro et al., 35 HPV-positive women—most of whom exhibited a CST IV status—received oral treatment with a probiotic (*L. crispatus* M247) for 90 days [65]. Following this period, a reduction of approximately 70% in HPV positivity was observed. There was also a significant change in CST status, with 94% of the women classified as CST I after 90 days of therapy [59]. Similar results were demonstrated in a study by Dellino et al. involving 160 women affected by HPV [60]. The women were randomly assigned to two groups: one receiving oral *L. crispatus* M247 (Group 1, *n* = 80) and a control group receiving only follow-up care (Group 2, *n* = 80). Over a median follow-up of 12 months, the total HPV clearance increased from 9.3% to 15.3% in patients taking long-term oral *L. crispatus* M247 compared to those who received no intervention [66].

Additionally, Palma et al. assessed 117 women with bacterial vaginosis (BV) or fungal infections accompanied by cytological changes or HPV presence [67]. Participants were divided into two groups and treated with a vaginal capsule of *L. rhamnosus* BMX 54 following their initial infection treatment. Group 1 (*n* = 60) used the probiotic for 3 months, while Group 2 (*n* = 57) used it for 6 months. Samples collected at 3 and 6 months indicated that the clearance of cytological changes was twice as high in Group 2. After 9 months, HPV clearance was also greater in Group 2 (31.2% vs. 11.6% in Group 1), and this group experienced lower relapse rates of vaginal infections.

## 8. Conclusions

Numerous studies mentioned in this review confirm the fact that the cervicovaginal microbiome plays a crucial role in maintaining genital tract homeostasis and significantly influences the risk of persistent HR-HPV infection and the development of cervical intraepithelial neoplasia. A *Lactobacillus*-dominated microbiota, particularly with *L. crispatus*, is associated with lower microbial diversity, effective immune regulation, and enhanced HPV clearance, whereas dysbiosis, characterized by reduced lactobacilli and increased anaerobic bacteria, correlates with HPV persistence and CIN progression. The study highlights that not all *Lactobacillus* species are equally beneficial; *L. iners* (CST III) does not produce protective D-lactic acid, and the inerolysin it secretes may disrupt the epithelial barrier, thereby increasing susceptibility to viral infections. What is more, alterations in the cervicovaginal microbiome contribute to carcinogenesis through modulation of local immune responses, chronic inflammation, epithelial barrier disruption, and oxidative stress. Growing evidence suggests that microbiome composition may serve as a valuable biomarker for CIN risk stratification and a potential target for future preventive and therapeutic strategies in cervical cancer. The use of oral probiotics or VMT interventions demonstrates a positive effect in treating BV, improving HPV clearance, and preventing the progression of cytological changes. Currently, the exact mechanism of action of probiotics in cervical cancer is not fully understood. Moving forward, it is necessary to conduct larger-scale clinical studies and longitudinal tracking.

## Figures and Tables

**Table 1:** Community State Types (CSTs) of The Cervicovaginal Microbiome (CVMB).

Community State Type (CST)	Dominant Species
I	*Lactobacillus crispatus*
II	*Lactobacillus gasseri*
III	*Lactobacillus iners*
IV	Anaerobic and microaerophilic bacteria including *Prevotella, Dialister, Atopobium, Gardnerella, Megasphaera, Peptoniphilus, Sneathia, Eggerthella, Aerococcus, Finegoldia i Mobiluncus.*
V	*Lactobacillus jenseni*

## Data Availability

No new data were created or analyzed in this study.

## Abbreviations

The following abbreviations are used in this manuscript:

CVMB	Cervicovaginal microbiome
HR	HPV High-Risk Human Papillomavirus
CIN	Cervical Intraepithelial Neoplasia
CC	Cervical Cancer
STIs	Sexually Transmitted Infections
HMP	Human Microbiome Project
HSIL	High-grade Squamous Intraepithelial Lesion
LSIL	Low-grade Squamous Intraepithelial Lesion
